# Rehabilitation of a Chronic Guillain-Barré Syndrome Patient With Vibratory Motor Stimulation of Dorsiflexors: A Case Report

**DOI:** 10.7759/cureus.53936

**Published:** 2024-02-09

**Authors:** Saurabh Agnihotri, Ankita Srivastava, Deepali Gupta, Fareha Naeem

**Affiliations:** 1 Department of Physiotherapy, College of Paramedical Sciences, Teerthanker Mahaveer University, Moradabad, IND; 2 Department of Physiotherapy, Teerthanker Mahaveer Hospital, Moradabad, IND; 3 Department of Physiotherapy, Teerthanker Mahaveer University, Moradabad, IND

**Keywords:** dorsiflexors, tonic vibration reflex, chronic guillain-barré syndrome, electric stimulation, exercise intervention, gbs, rehabilitation, vibratory motor stimulation

## Abstract

There are various reports describing physiotherapy rehabilitation in Guillain-Barré syndrome (GBS) but the use of current to rehabilitate GBS patients has remained an untouched topic. To elaborate on this work, we describe a case report focusing on the intervention plan for the rehabilitation of a chronic GBS case by the use of vibratory motor stimulation (VMS) current. The study aimed to describe the therapeutic application of VMS current in improving muscle power of dorsiflexors and overall outcome measures in a case of GBS presenting in a tertiary care hospital in North India.

A 29-year-old male patient came to Teerthanker Mahaveer University Hospital and consulted in the Department of Physiotherapy after 1.4 years of being diagnosed with acute motor axonal neuropathy-type GBS. Rehabilitation of this case included strengthening exercises of the upper and lower limbs along with balance exercises. Specifically, in this case, we gave VMS current after assessing the muscle power of the dorsiflexors, which was found to be grade-0 over the bilateral dorsiflexors, combined with passive dorsiflexion. Different outcome measures were used for assessment, including manual muscle testing, functional independence measurement, and the Berg Balance Scale. Improvement in the patient’s condition was observed in his outcome measures after two months of treatment.

There was an overall improvement in the muscle power of our patient’s dorsiflexors, where muscle power was upgraded from grade-0 to grade-I and grade-I+ in the bilateral lower limbs by the use of VMS current. This study marks a novel application of VMS to the dorsiflexors of a GBS patient, yielding positive outcomes in upgrading muscle power grades from grade-0 to grade-I and grade-I+. Further research is needed to confirm VMS efficacy as an early intervention in GBS patient rehabilitation.

## Introduction

Guillain-Barré syndrome (GBS) is an immune-based condition that presents with motor deficits (e.g., symmetrical ascending paralysis), acute polyneuritis, dysautonomia, and respiratory failure [1]. It is an immune-mediated peripheral neuropathy identified by quadriplegia, absence of deep tendon reflexes, paralysis of respiratory muscles, and dysautonomia. In its acute phase, approximately 25% of patients require ventilator support. Commonly recognized variants of GBS include acute motor axonal neuropathy (AMAN), acute inflammatory demyelinating polyneuropathy, Miller Fisher syndrome, and acute sensory and motor axonal polyneuropathy. 

The prevalence of GBS in India is similar to that of other developed countries (i.e., 1-2 patients/100,000 population with a male-to-female ratio of 2:1) [1]. GBS is the leading cause of muscle paralysis after poliomyelitis [2]. The mortality rate of GBS is 3.5%-12%. Almost 20% of GBS patients suffer from drastic disability, specifically in walking. The disease prognosis depends on a broad range of components, including preceding illness, advanced age, extent of disability, progression rate, and time of therapeutic intervention. Nerve conduction velocity testing and cerebrovascular tissue examination are commonly used for diagnostic confirmation, both of which can be normal in acute-phase GBS. The goals of GBS treatment are to hasten healing, lessen acute phase sickness consequences, and the lower risk of developing long-term neurological residual impairment.

Vibration to the skeletal muscles produces a contraction known as the tonic vibration reflex (TVR). Although there are various receptors stimulated on vibration, it is evident that the main receptor accounting for TVR is the primary ending of neuromuscular spindles [3]. Vibratory stimulation in adjunct with active effort was shown to evoke movement in patients with an inability to contract their impaired muscles. This approach is broadly used in physiotherapy and neurophysiology [4].

## Case presentation

A 29-year-old male patient had a paralysis attack on 15th August 2021 in his bilateral lower limbs and upper limbs, after which he was admitted to the Neurology Department of All India Institute of Medical Science in Rishikesh, India, where his blood samples were drawn and sent for analysis. After a complete blood count, lumbar puncture, thyroid stimulating hormone test, liver function test, nerve conduction velocity test, and electromyography, he was diagnosed with hypothyroidism and GBS. His medical treatment was immediately started, which included intravenous immunoglobulin injections (total 120 gm in four days) in adjunct with gabapentin, Neo-mercazole (10 mg three times per day), and propranolol (20 mg BD). The patient was discharged from the hospital after 23 days.

Six months later, the patient attended a follow-up appointment where he received advice to continue physiotherapy, which he diligently followed at home with the assistance of his brother. During this period, the patient experienced some improvement in his condition and began to turn to his side with support.

After one month, he followed up again and consulted with an endocrinologist and the Nuclear Medicine Department, where he was advised to continue carbimazole (10 mg three times per day). The patient underwent radioactive iodine therapy when his hypothyroidism was not controlled by medicine. Subsequently, the patient started taking Synthroid (50 mcg). The patient was then admitted to the Physical Medicine and Rehabilitation Department and remained there for 23 days for his physiotherapy rehabilitation.

On January 2, 2023, the patient consulted with the Physiotherapy Outpatient Department of Teerthanker Mahaveer University Hospital, where he was thoroughly assessed, and his rehabilitation was started. At that time, the patient continued on two medications- Rexite plus and Eltroxin 88mcg. Manual muscle testing (MMT) revealed grade-III responses of the bilateral shoulder flexors, bilateral shoulder extensors, bilateral shoulder abductors, bilateral shoulder adductors, bilateral shoulder external and internal rotators, bilateral elbow flexors and extensors, forearm pronators and supinators, and bilateral wrist flexors. Grade-II responses were seen for the bilateral wrist extensors and the radial and ulnar deviators. In the lower limbs, the bilateral hip flexors showed a grade-II response, the bilateral hip extensors showed a grade-I response, and the bilateral hip abductors, bilateral hip adductors, and bilateral hip internal and external hip rotators showed a grade-II response. The bilateral knee flexors showed a grade-I response, and the bilateral knee extensors showed a grade-II response. MMT for the bilateral ankle dorsiflexors and bilateral plantar flexors showed a grade-0 response. The Functional Independence Measure (FIM) and the Berg Balance Scale (BBS) were also used for the outcome measure. FIM, which included questions like bathing, dressing upper and lower body, toileting, etc., indicated a total score of 60/126. The BBS, including questions like sitting to standing, standing unsupported, etc., indicated a total score of 5/56. 

**Figure 1:**
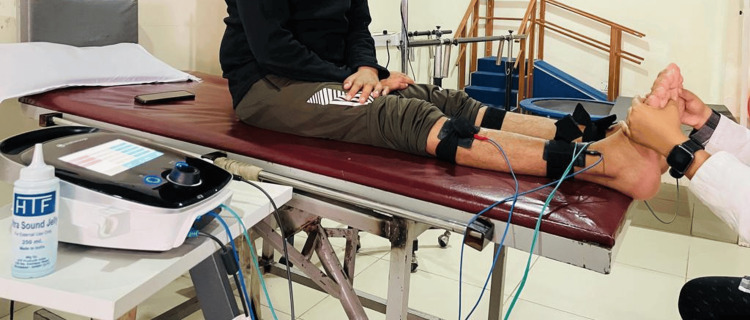
Application of vibratory motor stimulation over bilateral dorsiflexors

The patient’s rehabilitation program was scheduled every day from Monday to Saturday for 3 h per day, including rest periods. Exercise intervention included upper-limb exercises, as shown in Table 1, lower-limb exercises, as shown in Table 2, abdominal exercises, as shown in Table 3, balance exercises, fine motor skill exercises, and vibratory motor stimulation (VMS) over the bilateral dorsiflexors using a Chattanooga Intellect Mobile 2 Combo machine (parameters described in Table 4), as shown in Figure 1.

**Table 1:** Upper-limb exercises with the position of the patient and repetitions *REPS: Repetitions

Exercises	Position of the Patient	REPS/Sets
Active shoulder flexion	Sitting	10
Active shoulder extension	Sitting	10
Active shoulder abduction	Sitting	10
Active shoulder adduction	Sitting	10
Active elbow flexion	Sitting	10
Grip-strengthening with a smiley ball	Sitting	10
Peg board exercises	Sitting	10

**Table 2:** Lower-limb exercises with the position of the patient, repetitions, and sets *REPS: Repetitions

Exercises	Position of the Patient	REPS/Sets
Active assisted hip flexion exercise	Side lying	5/2
Active assisted hip extension exercise	Side lying	5/2
Active assisted hip abduction exercise	Supine lying	5/2
Active assisted hip adduction exercise	Supine lying	5/2
Assisted knee bending	Supine position	5/2
Passive ankle toe movement	Supine Position	5/2
Isometrics of abductors and adductors	Crook lying	5/2

**Table 3:** Abdominal exercises with the position of the patient, repetition, and sets *REPS: Repetitions

Exercises	Position of the Patient	REPS/Sets
Pelvic Bridging With Support	Crook lying	5/2
Abdominal crunches with support	Crook lying	5/2

**Table 4:** Vibratory motor stimulation current parameters

Characteristic of current	Value
Intensity	35 mA
Pulse duration	200 µs
Frequency	50 pps
Antifatigue	Off
Cycling	On
Rise of current	2.0 s
On time	5 s
Fall of current	2.0 s
Off time	5 s
Total Time	20 min

The patient first completed 19 sessions of passive dorsiflexors bilateral movement from 2nd Jan 2023 to 23 Jan 2023. Then, at the 20th session (25th Jan 2023), VMS was started over the bilateral ankle dorsiflexors. After 20 sessions of VMS current, flickering contractions over ankle dorsiflexors were noticed (grade-I), which further improved to grade-I+ after giving 10 more sessions. The VMS current parameters are shown in Table 4.

Balance exercises were introduced after two months, which included kneel standing with support, shoulder movements while kneel standing, sitting while balancing on a physioball, ball catching while sitting on a physioball, ball kicking in a sitting position, half knee standing with support of a stick, and picking up objects while kneel standing. There was appreciable improvement in the patient’s outcome measures. Functional independence measure (FIM) increased from 60/126 to 106/126, as shown in Table 5. The BBS increased from 5/56 to 37/56, as shown in Table 6. Improvement was also seen in MMT for the upper limbs, lower limbs, feet, and trunk muscles, as shown in Tables 7-10, respectively.

**Table 5:** Functional independence measure score comparison at initiation of physiotherapy (day 0) and after two months of follow-up

	At Initiation of Physiotherapy (Day 0)	Follow-up (After Two Months)
Self-care		
Eating	1/7	7/7
Grooming	1/7	4/7
Bathing	1/7	7/7
Dressing-upper body	1/7	7/7
Dressing-lower body	1/7	4/7
Toileting	1/7	4/7
Sphincter control		
Bladder management	7/7	7/7
Bowel management	7/7	7/7
Transfers		
Bed, Chair, Wheelchair	1/7	4/7
Toilet	1/7	7/7
Tub, Shower	1/7	7/7
Locomotion		
Walk/Wheelchair	1/7	6/7
Stairs	1/7	3/7
Motor Subtotal Score	25/91	71/91
Communication		
Comprehension	7/7	7/7
Expression	7/7	7/7
Social Cognition		
Social interaction	7/7	7/7
Problem- solving	7/7	7/7
Memory	7/7	7/7
Cognitive Subtotal Score	35/35	35/35
Total FIM Score	60/126	106/126

**Table 6:** Berg Balance Scale score comparison at initiation of physiotherapy and after two months of follow-up

Questions	At Initiation of Physiotherapy (Day 0)	After Two Months
Sitting to standing	0/4	3/4
Standing unsupported	0/4	2/4
Sitting with back unsupported but feet supported on floor or on a stool	3/4	4/4
Standing to sitting	2/4	4/4
Transfers	0/4	4/4
Standing unsupported with eyes closed	0/4	3/4
Standing unsupported with feet together	0/4	3/4
Reaching forward with outstretched arm while standing	3/4	0/4
Pick up object from the floor from a standing position	0/4	2/4
Turning to look behind over left and right shoulders while standing	0/4	2/4
Turn 360 degrees	0/4	2/4
Place alternate foot on step or stool while standing unsupported	0/4	2/4
Standing unsupported one foot in front	0/4	2/4
Stand on one leg as you can without holding on	0/4	1/4
Total=56	5/56	37/56

**Table 7:** Manual muscle testing score comparison of upper limbs before physiotherapy and after two months of follow-up Rt.=Right; Lt.=left

Muscle	Right (before physiotherapy)	Left (before physiotherapy)	Right (after two months of physiotherapy)	Left (after two months of physiotherapy)
Shoulder				
Flexor	3/5	3/5	5/5	5/5
Extensor	3/5	3/5	5/5	5/5
Abductors	3/5	3/5	5/5	5/5
Adductor	3/5	3/5	5/5	5/5
External Rotators	3/5	3/5	3/5	3/5
Internal Rotators	3/5	3/5	3/5	3/5
Elbow				
Flexors	3/5	3/5	5/5	5/5
Extensors	3/5	3/5	5/5	5/5
Forearm				
Pronators	3/5	3/5	5/5	5/5
Supinator	3/5	3/5	5/5	5/5
Wrist				
Flexors	3/5	3/5	4/5	4/5
Extensors	2/5	2/5	4/5	4/5
Radial Deviators	2/5	2/5	3/5	3/5
Ulnar Deviators	2/5	2/5	2/5	2/5

**Table 8:** Manual muscle testing score comparison of lower limbs before physiotherapy and after two months of follow-up Rt.=Right; Lt.=left

Muscles	Right (before physiotherapy)	Left (before physiotherapy)	Right (after two months of physiotherapy)	Left (after two months of physiotherapy)
HIP				
Flexor	2/5	2/5	4/5	4/5
Extensor	1/5	1/5	4/5	4/5
Abductors	2/5	2/5	4/5	4/5
Aductors	2/5	2/5	4/5	4/5
External Rotator	2/5	2/5	2/5	2/5
Internal Rotator	2/5	2/5	3/5	3/5
KNEE				
Flexors	1/5	1/5	2/5	2/5
Extensors	2/5	2/5	2+/5	2+/5

**Table 9:** Manual muscle testing score comparison of feet before physiotherapy and after two months of follow-up Rt.=Right; Lt.=left

Ankle	Right (before physiotherapy)	Left (before physiotherapy)	Right (after two months of physiotherapy)	Left (after two months of physiotherapy)
Dorsiflexor	0/5	0/5	1/5	1/5
Plantarflexor	0/5	0/5	1/5	1/5

**Table 10:** Manual muscle testing score comparison of trunk muscles before physiotherapy and after two months of follow-up Rt.=Right; Lt.=left

Muscles	Right (before physiotherapy)	Left (before physiotherapy)	Right (after two months of physiotherapy)	Left (after two months of physiotherapy)
Trunk Flexors	4/5	4/5	5/5	5/5
Trunk Extensor	4/5	4/5	5/5	5/5
Trunk Side flexor	4/5	4/5	5/5	5/5
Trunk Rotator	4/5	4/5	5/5	5/5

## Discussion

In this case report, we have thoroughly described the rehabilitation program (Videos 1, 2) of a chronic GBS patient. GBS is an autoimmune disorder where the peripheral nerves are attacked by the immune system; it is characterized by a sensation of pins and needles in the fingers, toes, ankle, or wrist; weakness, which is progressive from lower body to upper body; unsteady walking or inability to walk or climb stairs; difficulty with facial movements; severe pain; rapid heartbeat, low or high blood pressure; and difficulty breathing. After GBS is diagnosed, it must be treated early to avoid serious cardiac and pulmonary complications, as well as secondary infections. Patients typically recover well, with 60%-80% walking after six months [5].

**Video 1:** Rehabilitation of the chronic GBS case GBS: Guillain-Barré Syndrome

**Video 2:** Improvement in dorsiflexors

Lance from Prince Henry Hospital (Division of Neurology), University of New South Wales, Sydney, was the first to report the results of high-frequency, low-amplitude sinusoidal stretching on human skeletal muscles This stretching comprises an asynchronous involuntary motor unit contraction obtained by the application of mechanical vibration along with simultaneous reciprocal relaxation of the prime antagonist. Hagbarth and Eklund denoted this phenomenon as TVR and coined the term VMS to illustrate the mechanism of mechanical vibration producing TVR, or its correlates [3].

To date, TVR is primarily used in researching the usual functioning of neuromuscular spindles, the effects of numerous drugs targeting the central nervous system, and the pathologic physiology of neuromuscular disorders [3]. VMS produces an enormous emission of impulses from 1A afferents, which travel centrally to the spinal cord (dorsal root), synapse with alpha motor neurons, and transfer the signals again to the vibrated extrafusal muscle fibers, thereby evoking their contraction.

VMS is used therapeutically in neurological and non-neurological disorders in the field of physiotherapy. It can be combined with functional training or therapeutic exercise. VMS is also used as a research aid in assessing the effectiveness of methods that are known to facilitate or inhibit muscle action and as a tool in evaluating some neurological disorders [6]. Eklund and Hagbarth found that every skeletal muscle in humans was able to produce a TVR when vibrated. More significantly, when a paretic muscle was vibrated during a maximal voluntary effort, the contraction strength was higher than what would have been in the absence of the vibration.

In light of the well-known fact that TVR can be obtained from skeletal muscles despite them being normal, myotonic, spastic, or paretic [6], we introduced VMS current over our patient’s dorsiflexors and saw positive results after 20 sessions. The patient’s muscle power improved from grade-0 to grade-I, which was further enhanced after 10 sessions, upgrading the muscle power to grade-I+. Upon comparing the patient’s outcome measures before physiotherapy and after two months of treatment, there was considerable improvement across all outcome measures, including the FIM, BBS, and MMT.

## Conclusions

Although there are various case reports on rehabilitation in GBS patients, to the best of our knowledge, this is the first where VMS was given to the dorsiflexors of a chronic AMAN-type GBS patient, which produced positive results. Through this case report, we conclude that VMS current is effective in upgrading the muscle power of dorsiflexors from grade-0 to grade-I and grade-I+. Also there is a future scope that the treatment needs to be tested as an early intervention to conclude positively that VMS current is an effective modality in rehabilitation of GBS patients.
